# Cadmium disrupts hepatic lipid homeostasis: molecular mechanisms, unresolved controversies, and therapeutic strategies

**DOI:** 10.1016/j.isci.2025.114406

**Published:** 2025-12-11

**Authors:** Ruilong Li, Xuemeng Wang, Wanwan Liu, Mingjie Song, Tao Zeng, Cuili Zhang

**Affiliations:** 1 Institute of Toxicology, School of Public Health, Cheeloo College of Medicine, Shandong University, Jinan, Shandong 250012, China

**Keywords:** Biological sciences

## Abstract

Cadmium, a pervasive environmental toxicant with profound bioaccumulation potential, poses a significant threat to hepatic lipid homeostasis. This review systematically delineates the intricate molecular mechanisms underlying the cadmium-induced dysregulation of hepatic lipid metabolism. We highlight that cadmium accumulation in the liver triggers a cascade of pathological events, including mitochondrial dysfunction, aberrant activation of nuclear receptors driving *de novo* lipogenesis, and epigenetic reprogramming. Concurrently, cadmium exacerbates oxidative stress, amplifies inflammatory cascades, and disrupts the gut-liver axis. Critically, unresolved controversies such as the dual effects on liver lipid homeostasis under chronic environmental cadmium exposure, sexual dimorphism in susceptibility (potentially estrogen-mediated), and synergistic hepatotoxicity from co-exposure with microplastics are discussed. We further explore emerging therapeutic strategies targeting these pathways, including antioxidant therapy, epigenetic modulation, and microbiota-based interventions. This synthesis clarifies the mechanistic pathways linking cadmium exposure to hepatic lipid accumulation and identifies critical research gaps for future investigation.

## Graphical abstract

**Figure:**
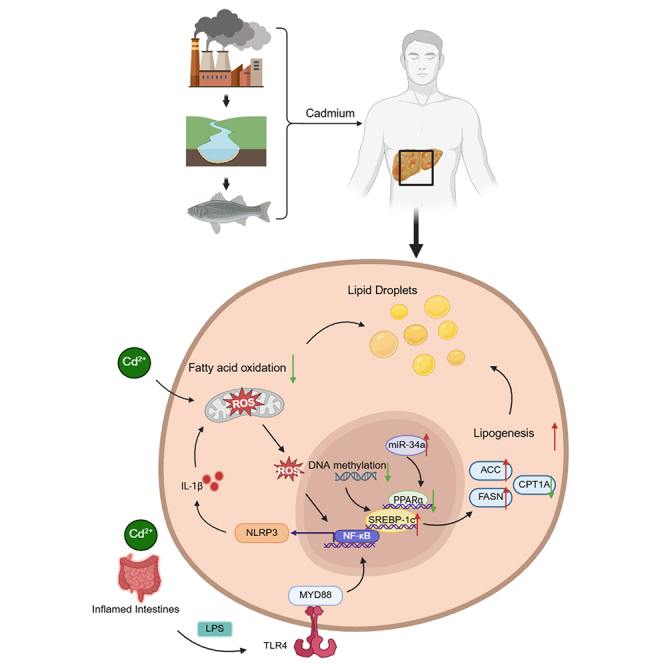

## Introduction

Heavy metals are a pervasive environmental contaminant that pose substantial acute and chronic health hazards to both occupational and general populations. Cadmium, a widespread environmental pollutant, primarily originates from industrial emissions, agricultural use of cadmium-containing fertilizers, and tobacco combustion. It exhibits strong bioaccumulation potential and a long biological half-life (10–30 years), entering the human body via the food chain and accumulating in organs such as the liver and kidneys.^1^ Cadmium disrupts proteostasis and oxidative balance across organs, targeting distinct molecular pathways. Renal toxicity involves impaired autophagy-lysosome function through transcription factor EB inhibition via mechanistic target of rapamycin complex 1/chromosome region maintenance 1 activation and general control non-repressed protein 5-mediated acetylation,^2^ and necroinflammation via MLKL-Drp1-mtROS signaling.^3^ In the brain, cadmium downregulates SIRT5, causing succinylation-dependent lysosomal Ras-associated protein 7a inactivation that blocks autophagosome-lysosome fusion, promoting Alzheimer’s-like amyloid pathology.^4^ Bone toxicity stems from AKT inhibition, triggering transcription factor E3 nuclear translocation and excessive autophagy-mediated bone mesenchymal stem cells death.^5^ These findings highlight organ-specific cadmium targets converging on dysregulated clearance mechanisms and redox imbalance.

The liver, as a primary target of cadmium toxicity, experiences disruption in lipid metabolic homeostasis, leading to abnormal lipid droplet accumulation, hepatic fibrosis, and even hepatocellular carcinoma.^6^ Epidemiological studies indicate that environmental cadmium exposure is closely associated with metabolic diseases, particularly a significant increase in the incidence of metabolic associated fatty liver disease (MAFLD).^7^ A cross-sectional survey indicates that higher blood cadmium exposure levels were associated with increased risks of hypercholesterolemia, hypertriglyceridemia, mixed hyperlipidemia, and high LDL-C (*p* < 0.05).^8^ With accelerating industrialization, cadmium pollution poses an escalating threat to human health. Elucidating its molecular mechanisms in disrupting lipid metabolism not only enhances understanding of the link between environmental pollution and metabolic diseases but also provides a theoretical foundation for developing targeted interventions.^9^

The mechanisms underlying cadmium-induced hepatic lipid metabolism dysregulation involve cross-talk among multiple pathways. Studies demonstrate that cadmium inhibits key enzymes of mitochondrial fatty acid β-oxidation (e.g., carnitine palmitoyltransferase-1A (CPT1A)) and activates lipid synthesis transcription factors such as sterol regulatory element-binding protein 1c (SREBP-1c), causing an imbalance between fatty acid synthesis and degradation.^10^^,^^11^ Additionally, cadmium-triggered oxidative stress activates the Jun N-terminal Kinase (JNK)/Nuclear Factor-κB (NF-κB) inflammatory pathway, exacerbating lipid accumulation in hepatocytes.^12^ Recent research further reveals that cadmium suppresses autophagy flux (e.g., downregulation of LC3-II/Beclin1) and disrupts epigenetic regulation (e.g., miR-34a-mediated SIRT1 inhibition), impairing lipid droplet clearance and accelerating MAFLD progression.^13^^,^^14^ Despite the fact that extensive research has confirmed that cadmium exposure disrupts hepatic lipid metabolism through mechanisms such as inducing oxidative stress, impairing fatty acid β-oxidation, and disrupting the peroxisome proliferator-activated receptor alpha (PPARα) signaling pathway, further investigation is required into its dose-dependent effects and the regulatory role of gender differences in cadmium toxicity. The prevailing focus of contemporary research endeavors concerning cadmium exposure and hepatic lipid homeostasis is predominantly oriented toward laboratory models. However, there is a conspicuous absence of research that delves into the underlying mechanisms and of data pertaining to ecotoxicological risk assessment.

This review aims to systematically elucidate the latest research advances in understanding how the environmental pollutant cadmium disrupts hepatic lipid metabolism homeostasis through cross-scale mechanisms involving oxidative stress, inflammatory signaling, epigenetic regulation, and gut-liver axis interactions. Beginning with an analysis of hepatic metabolic characteristics of cadmium and pathological associations with hepatotoxicity, we clarify the multi-level interaction networks triggered by its hepatic accumulation, including mitochondrial dysfunction, nuclear receptor (PPARγ/SREBP-1c)-mediated hyperactivation of lipid synthesis, epigenetic reprogramming, and gut microbiota-lipopolysaccharide (LPS)/farnesoid X receptor (FXR) axis disruption. Furthermore, we explore controversial research gaps such as the complex dose-response characteristic, gender disparities, and the synergistic effects of composite pollution, while proposing emerging translational strategies including targeted antioxidant therapies, epigenetic editing, and microbiota-based interventions. This synthesis provides theoretical foundations and promising research directions for preventing cadmium-induced hepatic lipid homeostasis imbalance.

## Hepatic metabolic characteristics of cadmium

**Figure 1:**
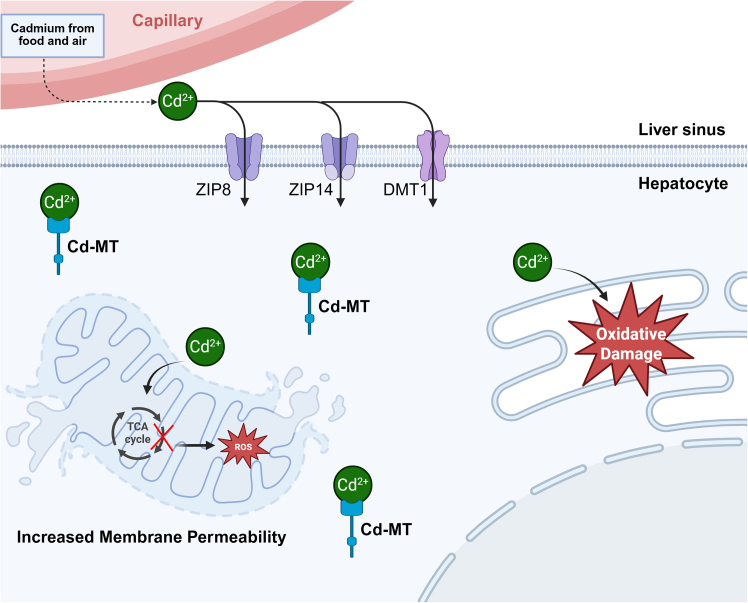
Cadmium absorption, distribution, and toxicity mechanisms in the liver

Cadmium enters the body via the digestive tract (dietary intake) or the respiratory tract (air pollution, smoking) and is transported to the liver through systemic circulation, making the liver a primary storage organ.^15^ Hepatic cadmium uptake involves multiple transporters, including divalent metal transporter 1 (DMT1) and zinc transporter proteins (ZIP8/14), which facilitate active cellular uptake.^16^ Inside hepatocytes, cadmium preferentially binds to metallothionein (MT) to form cadmium-MT complexes, reducing free cadmium toxicity and prolonging its half-life.^17^ However, when cadmium exposure exceeds MT-binding capacity, free cadmium accumulates in subcellular structures, particularly mitochondria and the endoplasmic reticulum (ER), where it disrupts calcium homeostasis and electron transport chain function, triggering oxidative damage (Figure 1).^18^

Lipid metabolism dysregulation is a hallmark of cadmium hepatotoxicity. In laying hens, cadmium exposure (60 mg/kg feed for 9 weeks) suppressed fatty acid β-oxidation enzymes (e.g., CPT1A, acyl-CoA oxidase 1) while upregulating fatty acid synthase (FAS) and SREBP-1c, leading to a 2.3-fold increase in hepatic triglycerides (TGs).^19^ Recent studies highlight critical biomarkers, including epigenetic and organelle-specific damage markers. Cadmium inhibits DNA methyltransferase (DNMT) activity, causing hypomethylation at promoters of lipid metabolism genes (e.g., PPARγ), thereby promoting aberrant expression.^20^ Additionally, endoplasmic reticulum (ER) stress markers glucose-regulated protein 78 (GRP78) and C/EBP-homologous protein (CHOP) increased 2.1- and 3.0-fold, respectively, in cadmium-exposed hens, implicating ER stress in hepatocytes.^19^ Notably, hepatocyte lipid droplet deposition, linked to the downregulation of lipid transport protein APOB100, has emerged as a biomarker in *Gobiocypris rarus* hepatocytes.^21^ Collectively, the metabolic process of cadmium in the liver provides a molecular basis for its disruption of hepatic lipid homeostasis.

## Oxidative stress and mitochondrial dysfunction

### Cadmium-induced ROS overproduction

Cadmium induces excessive ROS generation by impairing endogenous antioxidant systems (e.g., superoxide dismutase (SOD), GSH) and disrupting mitochondrial electron transport chain (ETC) function. Cadmium is a potent inhibitor of SOD activity and significantly depletes GSH levels. For example, in cadmium-exposed *Channa punctatus* (spotted snakehead), hepatic SOD and glutathione peroxidase (GPx) activities were significantly reduced, accompanied by GSH depletion, weakening ROS scavenging capacity.^22^ Similarly, in rat models, cadmium forms cadmium-GSH complexes by binding to sulfhydryl groups, depleting GSH reserves, and aggravating oxidative stress.^23^ Cadmium also inhibits glutathione reductase, disrupting GSH regeneration and further compromising antioxidant defenses.^24^

Moreover, cadmium disrupts mitochondrial ETC function to promote ROS production. In zebrafish (*Danio rerio*), Cd^2+^ displaces Fe^2+^ in iron-sulfur clusters, interfering with Complex I (NADH dehydrogenase) and Complex III (ubiquinol-cytochrome c reductase), leading to electron leakage and superoxide ion (O_2_^−^) generation.^25^ This disruption in rainbow trout (*Oncorhynchus mykiss*) manifests as reduced mitochondrial membrane potential and elevated ROS levels.^26^ Cadmium also induces inducible nitric oxide synthase overexpression, increasing NO production, which reacts with O_2_^−^ to form peroxynitrite (ONOO^−^), exacerbating mitochondrial damage.^18^ Cadmium further suppresses Complex IV (cytochrome *c* oxidase) activity, reducing ATP synthesis and increasing ETC electron leakage.^27^ In sheep models, cadmium exposure decreased hepatic mitochondrial respiratory enzyme activity, correlating with ROS accumulation and elevated MDA.^27^ Thus, cadmium establishes a vicious cycle of “oxidative stress-antioxidant depletion” through dual mechanisms, culminating in hepatic oxidative injury.^28^^,^^29^

### Mitochondrial collapse underlies the cadmium-induced disruption of hepatic lipid homeostasis

Mitochondrial impairment represents a primary initiating event in cadmium hepatotoxicity. Cadmium rapidly accumulates in mitochondria, directly inhibiting Complexes I/III, impairing β-oxidation, and triggering ROS bursts within hours of exposure. These defects constitute the earliest metabolic disruption, preceding overt steatosis. Mitochondrial membrane potential collapse is pivotal in cadmium-induced lipid dysregulation. Cadmium triggers mitochondrial permeability transition pore opening and Bax activation, reducing mitochondrial membrane potential. For example, in cadmium-exposed chickens (*Gallus gallus*), mitochondrial membrane integrity loss and ATP depletion coincided with ROS-mediated MDA elevation.^30^ Mitochondrial membrane potential collapse further inhibits fatty acid β-oxidation enzymes (e.g., CPT1A, ACADL). In mice, ETC dysfunction reduced NADH/FADH_2_ generation, limiting CoA and ATP supply for fatty acid oxidation.^31^ Cadmium also downregulates PPARα signaling, suppressing mitochondrial fatty acid transporter expression. For instance, in high-fat diet-fed mice, cadmium downregulated PPARα and CPT1A, promoting hepatic TG accumulation.^31^ Similarly, cadmium inhibits ACADL activity, blocking long-chain fatty acid β-oxidation. In Japanese flounder (*Paralichthys olivaceus*), cadmium activates mTORC1 to promote SREBP-1c nuclear translocation, upregulating FAS and acetyl-CoA carboxylase (ACC) expression.^32^ ER stress exacerbates lipid accumulation. In zebrafish co-exposed to cadmium and penthiopyrad, ER stress activates the inositol-requiring enzyme 1-X-box binding protein 1 signaling pathway, suppressing β-oxidation and enhancing lipogenesis.^25^ Additionally, cadmium inhibits mitophagy, leading to damaged mitochondrial accumulation and impaired fatty acid oxidation.^27^ These multi-pathway disruptions culminate in NAFLD-like pathologies.^29^^,^^33^ Thus, mitochondrial dysfunction acts as the core driver, propagating oxidative stress and energy depletion that activate inflammatory and transcriptional cascades.

### Dual-edged role of nuclear factor erythroid 2-related factor 2/antioxidant response element pathway activation

The nuclear factor erythroid 2-related factor 2/antioxidant response element pathway (Nrf2/ARE pathway) serves as a central defense mechanism against oxidative stress, yet chronic cadmium exposure manifests a dual-edged effect on its activation. Short-term cadmium exposure activates Nrf2 through Keap1-Nrf2 dissociation, thereby promoting the expression of antioxidant genes such as heme oxygenase-1 (HO-1), NADPH quinone dehydrogenase 1 (NQO1), and glutamate-cysteine ligase, catalytic subunit (GCLC). For instance, pretreatment with Moringa oleifera extract was shown to significantly attenuate cadmium-induced oxidative damage in hepatocytes by upregulating GPx1 and HO-1 via the Nrf2 signaling pathway.^24^ Similarly, selenium nanoparticles mitigated cadmium-triggered ferroptosis in hepatocytes by activating Nrf2 and enhancing glutathione metabolism.^29^

However, prolonged cadmium exposure leads to the hyperactivation of Nrf2 signaling, resulting in compensatory exhaustion. Studies in Nrf2-deficient mice demonstrated that chronic cadmium exposure exacerbates hepatic inflammation and fibrosis, underscoring the protective role of moderate Nrf2 activation.^33^ Paradoxically, sustained nuclear translocation of Nrf2 suppresses mitophagy, leading to the accumulation of damaged mitochondria and persistent ROS generation.^31^ Furthermore, Nrf2 activation may indirectly promote the expression of pro-inflammatory cytokines such as TNF-α and IL-6 by suppressing negative regulators of NF-κB, including IκB.^34^

The interplay between Nrf2 and lipid metabolism further complicates its biological effects. For instance, while Nrf2 activation suppresses SREBP-1c-mediated lipogenesis, its prolonged activation may disrupt PPARγ signaling, thereby inhibiting fatty acid oxidation.^35^ In sheep co-exposed to cadmium and molybdenum, Nrf2 hyperactivation induced the compensatory upregulation of mitochondrial antioxidant enzymes such as SOD2, yet paradoxically exacerbated lipid peroxidation.^27^ This metabolic disequilibrium indicates that sustained Nrf2 activation might shift from a protective to an injury-promoting role, particularly during chronic cadmium exposure.^33^^,^^36^ Table 1 provides a synopsis of the effects of cadmium exposure on antioxidant capacity and the Nrf2 signaling pathway under varying exposure models.

**Table 1:** The effects of cadmium on the antioxidant capacity and Nrf2 signaling pathway

Species	Cadmium Dose	Main Effects	Reference
Male ducks	50 mg/kg CdCl_2_ for 30 days	Cadmium exposure increased the contents of MDA and GSH, and decreased the activities of T-AOC, CAT and T-SOD.	Song et al.^37^
Female ICR mice	20 mg/kg CdCl_2_ for 4 weeks	GSH-PX content and SOD activity were clearly decreased in the Cd^2+^ group; The mRNA expression of HO-1, Nrf2 and NQO-1 was prominently decreased in cadmium-exposed liver tissues	Zhang et al.^38^
C57BL/6 mice	200 ppm cadmium for 24 weeks	Nrf2-KO mice exhibited significantly decreased levels of hepatic NRF2 downstream antioxidant genes and the mRNA levels of Mt1 and Mt2.	Chen et al.^33^
Male SD rats	2 mg/kg CdCl_2_ for 28 days	Compared with the control group, the SOD, CAT activities and GSH content diminished, and the MDA level accelerated in the livers of the cadmium groups, the mRNA expression of Nrf2, and NQO1 in the groups treated with cadmium diminished, while the mRNA expression of Keap1 increased.	Shi et al.^39^
Female fish	2.0 mg/L CdCl_2_ for 96 h	Compared with the control group, the levels of ROS and MDA were increased, the levels of GSH, GSH-Px and GST were decreased, the activity of SOD was ecreased. The mRNA expression of Nrf2 and its downstream target genes (HO-1, NQO1 and CAT) were decreased with the cadmium exposure. Keap1a and Keap1b mRNA expressions were upregulated.	Chen et al.^18^
Male C57BL/6 mice	100 mg/L CdCl_2_ for 30 days	MDA levels were elevated in the livers of mice after cadmium exposure, whereas the activities of CAT and SOD were decreased. WB results showed that Nrf2 protein levels were reduced, as well as the Kelch-like ECH-associated protein 1 (Keap1).	Zhang et al.^40^
Rats	5 mg/kg cadmium for 4 weeks	Cadmium exposure reduced the expression of Nrf2 and its cytoprotective genes (CAT, SOD, GPx, HO-1, GST and GSR) while upregulating Keap1 expression when compared with control rats.	Hayat et al.^41^
Male rats	5 mg/kg CdCl_2_ for 28 days	The treatment of cadmium alone decreased the levels of GSH and T-AOC as well as the activities of SOD, CAT and GSH-Px accompanied by the elevated level of MDA in the liver. The administration of cadmium alone decreased the mRNA and protein expressions of Nrf2, HO-1, NQO-1, GCLC, GCLM and GST in the liver.	Fang et al.^42^
HepG2 cells	15.6 μM CdCl_2_ for 6 h	Cadmium exposure reduces the expression levels of Nrf2, HO-1, and GPx1 in HepG2 cells, impairing their antioxidant capacity.	Buranasudja et al.^24^

## Cascade amplification of inflammatory

### Toll-like receptor 4/nuclear factor-κB pathway activation

Toll-like receptor 4 (TLR4)/NF-κB signaling amplifies cadmium toxicity as a secondary damage amplifier. Though cadmium weakly binds TLR4, its robust activation requires mtROS/DAMPs. Cadmium potentiates hepatic inflammatory responses by activating the TLR4 and its downstream NF-κB signaling pathway. TLR4, a critical receptor for recognizing pathogen-associated molecular patterns (PAMPs) and damage-associated molecular patterns (DAMPs), is activated by cadmium through mechanisms such as mimicking endogenous DAMPs (e.g., HMGB1) or directly binding to its extracellular domain.^43^ In cadmium-exposed broiler models, TLR4 expression was markedly upregulated, leading to NF-κB activation via the MyD88-dependent pathway and subsequent overproduction of pro-inflammatory cytokines TNF-α and IL-6.^44^ (Table 2, Row 1–2) Similarly, in rat livers, cadmium activated the TLR4/NF-κB axis, upregulated transcription of IL-1β and TNF-α, and triggered IκBα phosphorylation and degradation, thereby amplifying the inflammatory cascade.^45^ (Table 2, Row 3–5) NF-κB activation is closely linked to cadmium-induced perturbations in arachidonic acid metabolism. In C57BL/6 mice, cadmium elevated the activities of cyclooxygenase and lipoxygenase, promoting the synthesis of prostaglandin D2 and 20-hydroxyeicosatetraenoic acid. These metabolites further activated macrophages via the TLR4/NF-κB pathway, exacerbating hepatic inflammation.^43^ (Table 2, Row 6–7) Additionally, cadmium indirectly enhances NF-κB activity by suppressing the Nrf2 antioxidant pathway, which compromises cellular defenses against oxidative stress.^34^ (Table 2, Row 8) For example, in cadmium-exposed sheep models, inhibition of Nrf2 signaling resulted in sustained NF-κB activation and significantly elevated TNF-α and IL-6 levels.^46^ Preclinical studies demonstrate that inhibiting the TLR4/NF-κB pathway effectively mitigates cadmium-induced hepatotoxicity. The TLR4/NF-κB pathway involves numerous key molecules, which exhibit distinct patterns of change upon cadmium induction. The subsequent table provides a synopsis of the alterations and functions of these key molecules (Table 2). These findings underscore the TLR4/NF-κB axis as a key therapeutic target for cadmium-induced hepatic inflammation.

**Table 2:** Summary of key molecular alterations in cadmium-induced hepatic inflammation via the TLR4/NF-κB pathway

Molecule/Pathway	Change upon Cadmium Exposure	Function/Consequence	Experimental Model	Reference
TLR4	↑Expression/↑Activation (Direct binding to extracellular domain; Indirect activation via DAMPs e.g., HMGB1)	Pattern recognition receptor for PAMPs/DAMPs; Initiates inflammatory signaling	Broilers, Mice	Gong et al.^43^; Fan et al.^44^
MyD88	↑Recruitment/↑Activation (MyD88-dependent pathway)	Key adaptor protein downstream of TLR4; Transduces activation signal	Broilers	Fan et al.^44^
IκBα	↑ Phosphorylation/↑ Degradation	Inhibitory protein sequestering NF-κB in cytoplasm; Degradation releases active NF-κB	Rats	Sulayman Aboulqassim et al.^45^
NF-κB (p65 subunit)	↑ Activation/↑ Nuclear Translocation	Master pro-inflammatory transcription factor	Rats, Broilers	Sulayman Aboulqassim et al.^45^; Sarmiento-Ortega et al.^47^
Pro-inflammatory Cytokines	TNF-α↑, IL-6↑, IL-1β↑	Mediate hepatic inflammation, cell damage, and recruitment of immune cells	Broilers, Rats	Fan et al.^44^; Sulayman Aboulqassim et al.^45^; Zhang et al.^48^
COX/LOX Enzymes	↑ Activity	Key enzymes in arachidonic acid (AA) metabolism; Generate pro-inflammatory eicosanoids	Mice (C57BL/6)	Gong et al.^43^
PgD2/20-HETE	↑ Synthesis	Pro-inflammatory AA metabolites; Activate macrophages → ↑ TLR4/NF-κB signaling → Amplify inflammation and cytokine release	Mice (C57BL/6)	Gong et al.^43^
Nrf2 Signaling	↓ Activity/↓ Target gene expression (e.g., antioxidant enzymes)	Master regulator of antioxidant response; Suppression compromises cellular defense against oxidative stress, promoting inflammation	Sheep	Alruhaimi et al.^34^; Liu et al.^46^

### Kupffer cell polarization (M1 type dominant) exacerbates hepatocyte lipotoxicity

Kupffer cells (KCs), the liver-resident macrophages, play a pivotal role in cadmium-induced lipotoxicity through their polarization states (M1 pro-inflammatory or M2 anti-inflammatory phenotypes). Cadmium exposure drives KCs toward M1 polarization by activating the TLR4/NF-κB and mitogen-activated protein kinase (MAPK) pathways (JNK/p38). In cadmium-treated mouse livers, M1 markers (e.g., iNOS, CD86) were markedly upregulated, while M2 markers (e.g., Arg-1, CD206) were downregulated, resulting in the robust release of pro-inflammatory cytokines (TNF-α, IL-1β).^49^ This M1 polarization exacerbates hepatocyte lipid accumulation via paracrine mechanisms. For example, TNF-α secreted by M1-polarized KCs suppresses PPARα signaling in hepatocytes, impairing fatty acid β-oxidation, while simultaneously activating SREBP-1c to promote lipogenesis.^50^ M1-polarized KCs further aggravate hepatocyte injury by generating ROS and inflammatory mediators that directly compromise mitochondrial function. In cadmium-exposed chicken models, M1 polarization induced the collapse of mitochondrial membrane potential, reduced ATP synthesis, and elevated levels of lipid peroxidation products (e.g., MDA), ultimately triggering hepatocyte steatosis.^51^ These findings highlight the important role of cadmium in regulating hepatic lipid homeostasis through the modulation of KCs polarization.

### NOD-like receptor protein 3 inflammasome activation mediates pyroptosis

Cadmium exacerbates hepatic inflammation and lipid metabolic dysregulation by activating the NOD-like receptor protein 3 (NLRP3) inflammasome, which triggers hepatocyte pyroptosis. NLRP3 activation involves a two-step mechanism: cadmium first primes the transcription of NLRP3 and pro-IL-1β via the TLR4/NF-κB pathway (priming signal), followed by mitochondrial ROS and K^+^ efflux driving inflammasome assembly (activation signal).^22^ In cadmium-exposed chicken hepatocytes, upregulated expression of NLRP3, ASC, and Caspase-1 promotes the maturation and release of IL-1β and IL-18, while Gasdermin D (GSDMD)-mediated pore formation in the cell membrane initiates pyroptosis.^51^ Critically, this cadmium-triggered inflammatory cascade centrally disrupts hepatic lipid homeostasis through a potent signaling axis: Mature IL-1β and IL-18, released downstream of inflammasome activation, engage their cognate receptors to robustly activate both the JNK and NF-κB signaling pathways.^26^^,^^34^ This dual activation exerts a profound bidirectional impact on lipid metabolism: (1) It suppresses PPARα-mediated fatty acid β-oxidation, potentially through direct transcriptional repression and/or modulation of PPARα cofactors, thereby limiting lipid utilization for energy; and (2) Concurrently, it upregulates SREBP-1c-driven lipogenesis. Activated SREBP-1c translocates to the nucleus and transcriptionally enhances the expression of key lipogenic enzymes, promoting *de novo* fatty acid synthesis. This concerted suppression of lipid catabolism and stimulation of lipid anabolism creates a potent pro-lipogenic environment, culminating in hepatic lipid accumulation. These findings provide a mechanistic rationale for targeting the NLRP3 pathway in treating cadmium-associated liver diseases with comorbid lipid metabolic disorders. Inflammation thus serves as a critical amplification loop, extending cadmium’s primary mitochondrial effects into sustained metabolic failure.

## Dysregulation of nuclear receptors and key enzymes in lipid metabolism

### Peroxisome proliferator-activated receptor gamma overexpression promotes adipogenesis

Peroxisome proliferator-activated receptor gamma (PPARγ) is a key nuclear receptor regulating adipogenesis and lipid storage. Cadmium exposure induces PPARγ overexpression through multiple pathways, thereby promoting hepatic lipid accumulation. Studies have demonstrated that cadmium indirectly upregulates PPARγ expression by activating oxidative stress and inflammatory signaling pathways, such as the NF-κB/NLRP3 inflammasome axis. For instance, in a cadmium-exposed rat model, both mRNA and protein levels of PPARγ were significantly elevated, accompanied by increased hepatic triglyceride and free fatty acid content.^52^ Furthermore, cadmium exacerbates lipogenesis by suppressing AMPK activity, thereby abolishing the AMPK-mediated negative regulation of PPARγ.^53^ Activation of PPARγ directly promotes the expression of lipid synthesis-related genes, including FASN and ACC. *In vitro* experiments revealed that cadmium-treated hepatocytes exhibited enhanced binding activity of PPARγ to the promoter regions of lipogenic genes, leading to the abnormal activation of lipid synthesis pathways.^54^ Additionally, the synergistic interaction between PPARγ and SREBP-1c amplifies lipid accumulation. For example, cadmium upregulates both PPARγ and SREBP-1c, increasing fatty acid synthase activity and ultimately causing lipid droplet deposition in hepatocytes.^55^ Notably, PPARγ overactivation is also associated with insulin resistance, establishing a vicious cycle of “lipotoxicity-metabolic dysregulation.”^56^ Intervention studies indicate that PPARγ inhibition significantly alleviates cadmium-induced hepatic steatosis. Farnesol, for instance, improves hepatic lipid metabolism by upregulating PPARγ antagonists and suppressing lipogenic gene expression.^52^ In conclusion, PPARγ overexpression represents a central mechanism underlying cadmium-induced hepatic lipid metabolism disorders.

### AMPK/mTOR/sterol regulatory element binding protein-1c signaling dysregulation drives lipogenic imbalance

SREBP-1c activation functions as the dominant transcriptional effector executing lipogenesis. While modulated by cadmium, its proteolytic cleavage and nuclear translocation primarily occur secondary to upstream insults. Cadmium disrupts hepatic lipid homeostasis by targeting the integrated AMPK/mTOR/SREBP-1c signaling axis. Central to this dysregulation is the suppression of AMPK activity, which abolishes the AMPK-mediated inhibition of mTORC1 and induces the dephosphorylation of downstream targets such as ACC, thereby promoting malonyl-CoA accumulation and suppressing fatty acid oxidation.^57^ Concurrently, cadmium hyperactivates mTORC1 through both AMPK-dependent and -independent mechanisms (e.g., via AKT/GSK3β insulin signaling), leading to dual lipogenic effects: (1) direct phosphorylation of SREBP-1c to enhance its nuclear translocation, upregulating FASN and ACC expression^58^^,^^59^; (2) suppression of autophagy flux (e.g., degradation of P62 inhibited), reducing lipid droplet degradation.^60^

SREBP-1c aberrant activation constitutes a critical effector. Cadmium induces ROS generation, upregulates ER stress markers (e.g., GRP78, CHOP), and facilitates SREBP-1c cleavage. Experimental evidence indicates that SREBP-1c inhibition reverses cadmium-induced lipotoxicity.^61^ Knockdown of SREBP-1c via siRNA significantly suppresses the cadmium-triggered upregulation of FASN and ACC, accompanied by reduced hepatic triglyceride content.^62^ Furthermore, cadmium modulates miRNA networks (e.g., miR-182-5p) to indirectly regulate SREBP-1c activity. Overexpression of miR-182-5p inhibits the TLR4/NF-κB signaling pathway, thereby reducing SREBP-1c nuclear translocation.^61^ Intervention studies reveal that AMPK agonists (e.g., AICAR) restore lipolytic capacity and reduce triglycerides by reactivating AMPK, upregulating β-oxidation genes (e.g., CPT1A), and counteracting mTOR-driven lipogenesis.^57^^,^^63^ Critically, SREBP-1c operates as a central downstream hub, integrating stress signals to upregulate lipogenic genes (FAS, ACC)—making it a potent therapeutic target, though not the initial trigger. Table 3 illustrates the alterations and regulatory effects of SREBP-1c and lipid metabolism-related genes in cadmium exposure studies conducted in both *in vivo* and *in vitro* experiments.

**Table 3:** Cadmium exposure leads to upregulation of SREBP-1c expression and increased lipid synthesis

Research Subjects	Gene Expression Changes	Alterations in Lipid Metabolism	Reference
Male Wistar rats	SREBP-1c↑	ORO staining for neutral lipid droplets corroborated triglyceride accumulation. The cadmium group displayed 3-fold more cells with great fatty deposits by field compared to the control group.	Sarmiento-Ortega et al.^64^
HepaRG cells	SREBP-1c, FABP, ACC, PPARγ↑	Cadmium even at 5 and 10 nM concentrations increased cell steatosis and upregulated the mRNA expression levels of SREPB1, ACC, and PPARG in HepaRG cells.	Niture et al.^58^
Male Wistar rats	SREBP1, SREBP2, FAS, HMGCOA↑ PPARα, CPT1, CPT2↓	Hepatic levels of CHOL and TGs were significantly increased in the livers of CdCl_2_-treated rats as compared to control rats and the liver architectures of the CdCl_2_-treated rats showed severe accumulation of lipid vacuoles of all sizes.	Alshehri et al.^17^
C57BL/6 mice	SREBP-1, FAS, SCD↑ ATGL, CPT1, LPL↓	The levels of TG and TC in serum and liver were increased by cadmium and the result of Oil red O staining confirmed that cadmium exposure significantly caused hepatic lipid accumulation.	Wan et al.^12^

## Epigenetic regulatory mechanisms

### DNA methylation and histone modifications

Epigenetic modifications (e.g., SREBP-1c promoter hypomethylation) act as stabilizers of pathological phenotypes. Induced by early oxidative stress, they perpetuate lipogenic gene expression beyond initial cadmium exposure. Cadmium exposure disrupts hepatic lipid homeostasis by altering DNA methylation patterns to modulate lipid metabolism-related gene expression. Studies reveal that cadmium activates lipogenic genes through DNA hypomethylation in hepatocytes. For instance, cadmium exposure significantly reduces methylation levels in the promoter region of the nuclear receptor PPARγ, enhancing its transcriptional activity and thereby upregulating FAS and SREBP-1c expression to accelerate lipogenesis.^65^ Furthermore, long-term cadmium exposure (1 year) induces genome-wide methylation alterations in rat liver tissues, with the aberrant methylation of metabolic pathway genes (e.g., MAPK, PI3K-Akt) strongly correlating with lipid metabolism disorders.^66^ Cadmium also disrupts epigenetic homeostasis by suppressing DNA methyltransferase (DNMT) activity. In Oreochromis niloticus (Nile tilapia), cadmium downregulates DNMT3a and DNMT3b expression, causing global DNA hypomethylation in the liver alongside elevated oxidative stress markers (e.g., malondialdehyde (MDA), 8-hydroxy-2′-deoxyguanosine (8-OHdG)), which exacerbates lipid peroxidation.^67^ Similarly, the cadmium-induced hypomethylation of the F2rl3 gene promoter in rat hepatocytes activates inflammatory signaling (e.g., NF-κB), promoting lipid deposition and hepatic fibrosis.^65^ These findings collectively demonstrate that cadmium disrupts the equilibrium between lipid synthesis and catabolism by targeting methylation modifications of metabolism-associated genes.^68^

Cadmium exposure disrupts lipid metabolism by reprogramming histone modification patterns to modulate the transcription of metabolic genes. Research demonstrates that cadmium elevates histone H3 lysine 9 acetylation levels in hepatocytes, which activates promoter regions of lipogenic genes (e.g., ACC, SCD1) to promote *de novo* fatty acid synthesis.^69^ Furthermore, cadmium suppresses histone deacetylase activity, leading to the hyperacetylation of histone H4 lysine 16 and enhanced transcriptional activity of SREBP-1c, thereby exacerbating hepatic steatosis.^70^ Cadmium also perturbs lipid homeostasis through histone methylation modifications. For example, cadmium upregulates the histone methyltransferase EZH2, increasing H3 lysine 27 trimethylation to repress fatty acid oxidation genes (e.g., CPT1A).^71^ Concurrently, cadmium activates the JNK signaling pathway to induce histone H3 serine 10 phosphorylation, which stimulates pro-inflammatory cytokine release (e.g., TNF-α) and indirectly aggravates lipid dysregulation.^72^ These epigenetic alterations collectively remodel the chromatin accessibility landscape, driving transcriptional reprogramming of lipid metabolism-associated genes.^69^

### MicroRNA regulatory network

MicroRNAs (miRNAs) play a central role in cadmium-induced hepatic lipid homeostasis. For instance, cadmium upregulates miR-21 to suppress nuclear factor erythroid 2-related factor 2 expression, impairing the antioxidant defense system and exacerbating oxidative stress-driven lipid peroxidation.^73^ Simultaneously, miR-21 inhibits fatty acid β-oxidation by targeting PPARα and CPT1 genes, resulting in hepatic triglyceride accumulation.^73^ Another critical miRNA, miR-34a, aggravates lipogenesis by suppressing the SIRT1/FXR signaling axis, thereby upregulating SREBP-1 and FAS expression.^17^ Cadmium also facilitates interorgan crosstalk via exosome-mediated miRNA transfer. Hepatic-derived exosomes carrying the long non-coding RNA MT1DP sequester miR-214, relieving its suppression on Bcl-xL to promote renal apoptosis, which indirectly amplifies hepatic lipotoxicity.^74^ Additionally, cadmium upregulates miR-155 to inhibit the Rheb/mTOR pathway, triggering aberrant autophagy and impairing lipid droplet degradation.^75^ Collectively, these miRNA networks orchestrate multi-target regulation of lipid metabolism hub molecules, forming an intricate pathogenic cascade.^76^

## Gut-liver axis crosstalk

### Cadmium disrupts gut barrier integrity

Cadmium disrupts hepatic lipid metabolism by directly impairing intestinal barrier function and increasing gut permeability, thereby facilitating endotoxin translocation (e.g., lipopolysaccharide, LPS) to the liver. Gut-derived signals (LPS/FXR dysregulation) operate as extrinsic propagators. Cadmium-induced intestinal barrier breach enables endotoxin influx, synergizing with intrahepatic drivers to potentiate inflammation and lipotoxicity. The integrity of the intestinal barrier relies on tight junction proteins (e.g., ZO-1, Occludin) and the mucus layer secreted by goblet cells. Studies demonstrate that cadmium exposure significantly suppresses the mRNA and protein expression of ZO-1 and Occludin in murine intestines while reducing mucin secretion, collectively compromising intestinal barrier integrity.^77^ Furthermore, cadmium induces oxidative stress (e.g., ROS/MAPK pathway activation) to promote intestinal epithelial apoptosis, exacerbating barrier dysfunction.^78^ Following barrier disruption, translocated LPS enters the liver via the portal circulation and binds to TLR4 on hepatocytes, activating the NF-κB and NLRP3 inflammasome pathways. Cadmium-exposed mice exhibit upregulated hepatic TLR4 and MyD88 expression alongside elevated pro-inflammatory cytokines (e.g., IL-1β, TNF-α, IFN-γ).^77^ The LPS-TLR4 axis not only directly induces hepatocyte inflammation but also suppresses lipid catabolism by downregulating key regulators such as PPARα, leading to fatty acid oxidation impairment and lipid accumulation.^79^ Additionally, cadmium alters gut microbiota composition (e.g., reduced Lactobacillus and Bifidobacterium), weakening intestinal immune homeostasis and forming a self-perpetuating vicious cycle.^30^ Notably, cadmium-induced depletion of gut microbiota-derived metabolites, such as secondary bile acids, synergistically exacerbates LPS-mediated hepatotoxicity. For instance, cadmium inhibits intestinal bile salt hydrolase activity, causing tauro-conjugated β-muricholic acid (T-βMCA) accumulation. T-βMCA suppresses intestinal FXR signaling, indirectly amplifying hepatic inflammation.^21^ These findings collectively identify gut barrier disruption and LPS-driven hepatic inflammation as pivotal mechanisms underlying cadmium-induced lipid metabolic disorders.^80^

### Dysregulation of bile acid metabolism

Cadmium disrupts hepatic lipid homeostasis by impairing bile acid (BA) synthesis, transport, and signaling, primarily through the suppression of FXR activity. FXR activation normally inhibits cholesterol 7α-hydroxylase (CYP7A1) expression to limit BA synthesis. However, cadmium exposure markedly reduces ileal FXR and its downstream target FGF15 in mice, resulting in aberrantly elevated hepatic CYP7A1 activity and excessive BA production.^79^ Additionally, cadmium inhibits BA efflux transporters (e.g., BSEP, MRP2), impairing BA excretion and promoting intrahepatic BA accumulation.^81^ BA dysregulation further exacerbates hepatocellular oxidative stress and mitochondrial dysfunction. For instance, cadmium increases hepatic levels of hydrophobic BAs (e.g., deoxycholic acid, DCA), which activate the JNK pathway to induce hepatocyte apoptosis.^82^ Concurrently, BA accumulation disrupts the FXR-SHP signaling axis, attenuating its suppression of SREBP-1c, thereby upregulating lipogenic genes (e.g., ACC, FAS) and promoting lipid deposition.^78^ Gut microbiota critically modulate BA metabolism through bile salt hydrolase activity. Cadmium exposure depletes Bacteroides species capable of BA deconjugation, inhibiting the conversion of primary to secondary Bas.^83^ This dysbiosis not only diminishes FXR activation but also promotes pro-inflammatory cytokine release via the Takeda G protein-coupled receptor 5 pathway.^84^ Furthermore, intestinal barrier dysfunction allows unconjugated BAs to enter the liver, amplifying hepatocyte injury through membrane destabilization.^85^

### Gut microbiota dysbiosis and short-chain fatty acid reduction

Cadmium exposure reshapes gut microbiota composition, characterized by an elevated Firmicutes/Bacteroidetes (F/B) ratio and reduced short-chain fatty acid (SCFA) production, exacerbating hepatic lipid homeostasis imbalance via the gut-liver axis. Studies reveal that cadmium decreases the abundance of SCFA-producing genera (e.g., Roseburia, Prevotella), leading to diminished levels of acetate, propionate, and butyrate.^83^ SCFAs normally suppress hepatic lipogenic enzyme activity through GPR41/43 receptor activation; their depletion directly promotes lipid accumulation.^86^ The increased Firmicutes dominance correlates with upregulated LPS biosynthesis genes, aggravating hepatic inflammation. For instance, cadmium elevates Proteobacteria abundance (e.g., *Escherichia coli*), enhancing endotoxin release.^87^ Concurrently, microbiota dysbiosis reduces bile acid metabolism enzyme activity (e.g., 7α-dehydroxylase), inhibiting secondary bile acid generation.^84^ Intervention studies demonstrate that probiotics (e.g., Lactobacillus plantarum BGAN8) or SCFA precursors can partially counteract cadmium-induced dysbiosis. L. plantarum BGAN8 secretes exopolysaccharides (EPS-AN8) to enrich butyrate-producing bacteria and restore SCFA levels.^77^ Melatonin modulates microbiota structure (e.g., increasing Akkermansia), enhances SCFA production, and improves lipid metabolism.^82^ Additionally, selenium-enriched polysaccharides suppress pathogenic bacterial proliferation, downregulate pro-inflammatory cytokines, and restore intestinal barrier integrity.^88^

## Controversies and contradictions in research

Studies on cadmium-induced imbalance in liver lipid homeostasis have revealed a remarkable phenomenon that warrants further investigation. The extant research has principally concentrated on investigating the effects of cadmium exposure patterns that significantly exceed environmental levels, which represent a pivotal trigger for disrupting hepatic lipid homeostasis. Such exposure has been shown to disrupt AMPK/PPARα-mediated fatty acid oxidation by inducing severe oxidative stress, mitochondrial dysfunction, endoplasmic reticulum stress, and inflammatory responses, while simultaneously upregulating SREBP-1c-driven lipid synthesis, ultimately driving hepatic steatosis and damage. However, emerging evidence suggests that under specific conditions of high-fat diet, environmental dose of cadmium exposure does not exacerbate lipid burden, but rather can alleviate high-fat diet-induced hepatic lipid accumulation.^89^ As discussed in 3.3, the Nrf2 pathway exhibits biphasic regulation under both acute and chronic cadmium exposure conditions: short-term cadmium exposure induces adaptive antioxidant responses, whereas sustained chronic exposure may lead to the depletion of antioxidant components, suppression of mitochondrial autophagy, and non-targeted effects, ultimately shifting toward a pro-oxidative state. These phenomena indicate that cadmium exerts distinct effects on hepatic lipid homeostasis and antioxidant capacity at different doses and exposure durations. This finding indicates that research investigating cadmium’s health hazards should consider its impact under chronic environmental exposure conditions. It is recommended that future research efforts concentrate on the examination of alterations in hepatic lipid homeostasis across a range of animal models that have been exposed to cadmium doses at environmental levels. It is submitted that such investigations will contribute to a deeper understanding of how environmental cadmium exposure influences the onset and progression of MAFLD.

Sexual dimorphism in cadmium toxicity modulation remains poorly characterized. Compared to female mammals, avian models such as ducks and geese offer reduced confounding from endogenous estrogen fluctuations due to their seasonal monovular reproductive pattern. While some experiments utilize female ducks in cadmium toxicity studies, the potential protective role of estrogen—via the regulation of lipid-metabolizing enzymes (e.g., CPT1A)^90^ or oxidative stress pathways—remains ambiguous. The prevalence of MASLD is higher in the general adult male population than in the female population. Furthermore, males are more susceptible to metabolic dysfunction-associated steatohepatitis (MASH), fibrosis, and liver-related complications. In clinical studies, young women with reproductive disorders characterised by altered estrogen levels or who underwent oophorectomy exhibited higher MASLD prevalence than young women of reproductive age.^91^^,^^92^ These findings suggest that the influence of sex hormones on hepatic lipid homeostasis is highly correlated with cadmium-induced lipid metabolism imbalance. The potential interaction between sex hormones and cadmium-induced lipid homeostasis disruption in animal models of cadmium exposure remains to be elucidated. Consequently, future studies should comprise samples from both sexes and measure hormone levels to ascertain whether sex hormone pathways play a role in cadmium-exposed animal models. However, most studies either neglect sex stratification or omit hormonal profiling,^93^^,^^94^ limiting mechanistic insights into gender-specific susceptibility.

Although research on cadmium-MPs co-exposure is expanding, critical uncertainties persist in mechanistic understanding and ecological risk assessment. Accumulating evidence suggests that cadmium-MPs synergy exacerbates hepatic oxidative damage and lipid accumulation via the “Trojan horse effect”—enhancing intestinal cadmium absorption while disrupting gut barrier integrity and microbiota homeostasis.^87^^,^^95^ The general population primarily encounters cadmium from ecological environments, thus making the ecological risk assessment of cadmium critically important. The bioaccumulation effects across trophic levels are pivotal to the evaluation of the ecological risks posed by composite pollution from MPs-cadmium. However, extant research has predominantly concentrated on detecting accumulation levels in individual species (e.g., fish),^96^ thereby overlooking the potential of MPs to modify cadmium migration pathways within food webs through a process termed “carrier-mediated cascade transfer.” This alteration has the capacity to shift the accumulation sites of cadmium carried by MPs within experimental animals, thereby affecting the risk assessment of MPs-cadmium complex accumulation in edible fish tissues. Consequently, future studies should utilize multispecies cascade experiments in an “algae → zooplankton → fish” exposure system to quantify the impact of MPs on cadmium bioaccumulation factors. Concurrently, the use of ^115^Cd-labeled MPs-cadmium complexes is recommended for the tracking of their migration pathways within food webs. A foundation must be established upon which a revision of the safety standards for cadmium in aquatic food products can be undertaken. Table 4 provides a synopsis of the collective exposure to cadmium and microplastics in diverse experimental animal models as documented in the extant literature, along with the principal effects of such combined exposure on the experimental models.

**Table 4:** Published articles reporting the effects of co-exposure of cadmium and MPs

Species	Co-exposure	Main Effects	Reference
Nematode	Cadmium (0.5 mg/L) PS-NPs (1 mg/L)	Nanoplastics promoted Cadmium storage in gut granules by forming GSH-Cd complexes.	He et al.^97^
male Kunming mice	CdCl_2_·2.5H_2_O (5 mg/kg) MPs (1 mg/d)	Co-exposure to cadmium and microplastics causes significant intestinal damage in mice, disrupts intestinal barrier integrity, promoting cancer cell proliferation. Proteomic analysis shows upregulation of SP1 and ABC transporters in co-exposure group.	Yang et al.^98^
Male BALB/c mice	Cadmium (100 mg/L) MPs (1 mg/L)	Combined exposure may intensify cell death, alter matrix tissue, and accelerate cell proliferation.	Li et al.^99^
Primary hepatocytes of C57BL/6 male mice	Cadmium (1.2 mg/L) NPs (10 mg/L)	NPs amplified the hepatocyte toxicity of Cd^2+^. NPs synergized with Cd^2+^ to induce more severe pyroptosis and apoptosis by activating the inflammatory caspase-1-dependent and Ca^2+^-mitochondrial-caspase-3 pathways to a greater extent, respectively.	Li et al.^1^
White feather broilers	Cadmium (140 mg/kg) MPs (100 mg/L)	MPs and Cadmium induced intestinal damage and liver inflammation in broilers by interfering with the TLR4/MyD88/NF-κB pathway and intestinal flora homeostasis.	Fan et al.^44^
C57BL/6 mice AML12 cells	Cadmium (50 mg/L) PS-MPs (10 mg/L)	The concurrent exposure of PS-MPs and Cd resulted in the blockage of hepatic lipid accumulation and autophagic pathway and further aggravated the toxic damage to the liver.	Chen et al.^96^
Male Kunming mice	CdCl_2_·2.5H_2_O (4.8 mg/kg/d) MPs (10.0 mg/d)	Exposure to cadmium and microplastics significantly perturbs the intestinal microbiome and metabolic profiles of mice, altering the metabolites associated with ABC transporters and the PPAR pathway.	Chen et al.^100^
Female Muscovy ducks	Cadmium (50 mg/kg) PVC-MP (1 mg/L)	Co-exposure significantly enhanced cadmium bioaccumulation, which may exacerbate the combined toxicity of PVC-MP and Cd in female ducks. Furthermore, co-exposure of PVC-MP with cadmium may induce hepatic glycolipid accumulation through the PCK1-PI3K/AKT signaling axis, which may subsequently lead to hepatic fibrosis.	Chen et al.^101^
BALB/c mice	Cadmium-rice (0.50 μg/g) MPs (2, 20, and 200 μg/g)	Microplastic co-exposure caused 1.17- to 1.38-fold higher cadmium accumulation in mouse tissue. Gut microbiota and gut metabolites were altered with microplastic co-exposure.	Chen et al.^102^
Procypris merus (fish)	Cadmium (500 μg/L) MPs (500 μg/L)	MPs enhanced cadmium accumulation in both the liver and gills of Procypris merus. Co-exposure affects lipid metabolism and potentially harms the health of Procypris merus.	Cheng et al.^95^

The exhaustive analysis presented in the preceding sections reveals a complex landscape of cadmium-induced hepatic lipid homeostasis imbalance. However, as highlighted by contemporary toxicological frameworks, mechanistic claims demand rigorous evaluation of evidence robustness. Here, we establish a tiered evidence assessment system (Table 5), critique model limitations, and identify key translational knowledge gaps. This critical appraisal highlights that while core pathways, such as oxidative stress and TLR4 activation, are mechanistically plausible, their environmental relevance demands verification in advanced chronic environmental dose exposure models incorporating human tissue biomimetics and multi-omics approaches.

**Table 5:** Strength of evidence for major pathways in cadmium-induced hepatic lipid homeostasis imbalance

Pathway	Core Mechanisms	Key Evidence	Evidence Strength	Limitations and Knowledge Gaps
Oxidative stress and mitochondrial dysfunction	Cadmium inhibits SOD/GSH; disrupts ETC complexes; suppresses CPT1A/β-oxidation	Replicated in rats/chickens/zebrafish^10^^,^^19^^,^^25^	Strong	Limited environmental cadmium exposure data
TLR4/NF-κB inflammatory axis	Cadmium activates TLR4→NF-κB→TNF-α/IL-6↑	Validated in mice/rats/chickens^43^^,^^44^^,^^45^; Human hepatocytes (HepG2)^24^	Robust	Human tissue data lacking; Evolutionary divergence in aquatic models (e.g., zebrafish TLR4 signaling)
NLRP3 inflammasome activation	Cadmium→ROS→ NLRP3→IL-1β/GSDMD →pyroptosis	Consistent in mammals (mice/chickens)^26^^,^^51^; Primary human hepatocytes^1^	Robust	Pyroptosis relevance at environmental doses unclear; No human chronic exposure data
Gut-Liver axis disruption	Cadmium damages gut barrier→LPS ↑; Dysbiosis→SCFA ↓	Confirmed in mice/ducks^77^^,^^101^;	Robust	Human microbiome data scarce
PPARγ/SREBP-1c lipogenic pathway	Cadmium ↑ PPARγ→FAS/ACC ↑; Inhibits AMPK→activates SREBP-1c	Replicated in rats/mice^52^^,^^58^; Human hepatocytes (HepRG)^58^	Moderate	Human *in vivo* validation absent; Sex differences (estrogen modulation) understudied
Epigenetic regulation	DNA hypomethylation (↑PPARγ); miR-34a↑ →SIRT1↓	Rat whole-genome methylation^66^; *In vitro* miR-34a mechanism^17^	Moderate	Human epigenetic data sparse; Limited *in vivo* functional validation

## Intervention strategy

Cadmium-induced hepatic lipid metabolism disorders, mediated by oxidative stress, inflammatory cascades, mitochondrial dysfunction, and gut microbiota dysbiosis, necessitate multi-target interventions integrating antioxidant, anti-inflammatory, metabolic regulatory, and microbiota-modulating approaches. Pharmacological agents such as *N*-acetylcysteine (NAC) alleviate cadmium hepatotoxicity by restoring GSH levels, suppressing lipid peroxidation, and stabilizing mitochondrial membranes via transcriptional activation of mitochondrial transcription factor A to regulate choline metabolism,^103^^,^^104^ while melatonin counters non-alcoholic fatty liver disease progression by inhibiting mitochondrial ROS, restoring autophagic flux (e.g., lipophagy), and rebalancing bile acid metabolism.^82^^,^^105^ Natural products, including polysaccharides and pomegranate peel extract, demonstrate dual detoxification effects through cadmium chelation, immune enhancement, and synergistic suppression of inducible nitric oxide synthase when combined with NAC.^106^^,^^107^ Quercetin, a naturally occurring polyphenol found in plants, has been shown to impede the progression of cadmium chloride-induced hepatic steatosis. This effect is primarily attributed to its capacity to suppress the upregulation of microRNA-21 by increasing the expression of Nrf2.^73^ Emerging combination strategies (e.g., NAC-melatonin co-administration, probiotic-polysaccharide synergism) highlight the potential of dual-pathway targeting (“gut detoxification-liver repair”), yet challenges persist in optimizing natural product bioavailability, ensuring biosafety of genetically modified strains, and translating multi-omics insights (metabolomics, metagenomics) into personalized therapies against chronic environmental levels of cadmium exposure.

## Human relevance and translational gaps

Epidemiological studies consistently demonstrate positive correlations between urinary/blood cadmium levels and MAFLD prevalence, serum triglyceride elevation, and altered cholesterol metabolism across diverse populations.^8^ Clinical investigations utilizing liver biopsies further reveal cadmium accumulation correlating with steatosis severity and dyslipidemia markers.^108^ However, most studies are cross-sectional, hindering causal inference; and confounding factors (e.g., co-exposure to other metals, diet) are inadequately controlled.

Based on the clues discovered by population research, the mechanism of cadmium-induced liver lipid metabolism imbalance is mostly studied using animals. Translating mechanistic insights from animals to human applications faces significant hurdles. Species divergence is critical: murine lipid metabolism differs substantially from humans, potentially overestimating cadmium’s impact on certain pathways (e.g., PPARα suppression may have greater functional consequences in rodents).^109^ Disease model limitations arise as most preclinical studies employ subacute cadmium exposures, failing to recapitulate chronic environmental exposure dynamics of humans. This obscures dose-response extrapolation and thresholds for adversity.

Therefore, prioritizing human-centric approaches is essential. Leveraging biobanks with paired metal biomonitoring, lipidomics, and genomic data can identify exposure-gene-metabolite interactions in large cohorts.^99^ Advanced *in vitro* models (e.g., cadmium-exposed human primary hepatocytes, 3D liver organoids, or multi-cell type spheroids) better capture human-specific toxicity pathways.^110^ Refined preclinical models should incorporate chronic environmental dose exposure regimens, humanized mice (e.g., expressing human PPARα),^111^ and comorbid conditions (e.g., diet-induced obesity). Finally, standardized exposure assessment and prospective cohort studies are needed to establish temporal relationships and dose-dependent effects in humans.

## Conclusion and future perspectives

**Figure 2:**
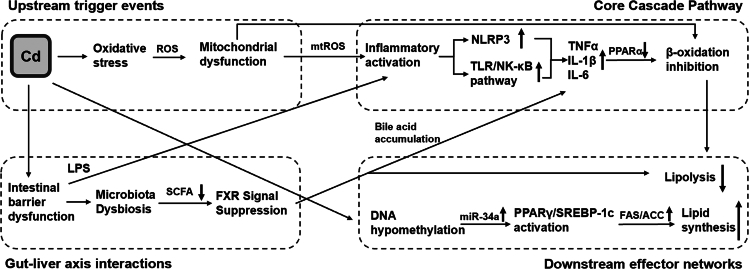
Integrated mechanistic network of cadmium-induced hepatic lipid homeostasis disruption. Cd^2+^ intake directly induces oxidative stress (ROS burst and antioxidant depletion), which acts as the upstream initiator of mitochondrial dysfunction and inflammation Mitochondrial damage suppresses fatty acid β-oxidation and amplifies ROS production. mtROS activates inflammatory pathways, including TLR4/NF-κB signaling and NLRP3 inflammasome assembly, leading to pro-inflammatory cytokine release. Cytokines and Cd^2+^ synergistically activate lipogenic transcription factors, upregulating FAS/ACC expression and promoting *de novo* lipogenesis. Cd^2+^-induced epigenetic modifications further enhance lipid accumulation by modulating SIRT1-PPARα/SREBP-1c axes. Cd^2+^ compromises intestinal barrier integrity, facilitating LPS translocation and hepatic inflammation. Gut dysbiosis reduces short-chain fatty acid (SCFA) production, impairing FXR signaling and bile acid homeostasis, which further exacerbates lipid metabolic dysfunction. TNF-α suppresses PPARα activity, creating a vicious cycle of impaired lipid catabolism. FXR inhibition promotes bile acid accumulation, which sustains inflammatory signaling.

While this review comprehensively details interconnected pathways in cadmium-induced hepatic lipid dysregulation, we further delineate their hierarchical roles based on mechanistic evidence. We distinguish primary drivers (direct cadmium effects initiating pathology) from secondary effectors/amplifiers (downstream executors or signal intensifiers) to clarify causal relationships. This review systematically elucidates the intricate and multi-layered mechanisms underpinning the cadmium-induced disruption of hepatic lipid metabolism, a critical process driving the development and progression of MAFLD (Figure 2). The evidence unequivocally demonstrates that cadmium accumulation within hepatocytes orchestrates a toxic cascade primarily through the concurrent induction of mitochondrial dysfunction (impairing β-oxidation via the suppression of CPT1A and ACADL), oxidative stress (via ETC disruption and antioxidant depletion), and robust inflammatory responses (fueled by the activation of TLR4/NF-κB and NLRP3 inflammasome pathways, coupled with M1 Kupffer cell polarization). It is imperative to note that cadmium exposure has been demonstrated to induce epigenetic reprogramming, manifesting as DNA hypomethylation, dysregulation of key microRNAs such as miR-34a, and histone modifications. This profoundly dysregulates the expression of central lipid metabolism regulators, notably PPARγ and SREBP-1c, thereby tipping the balance toward enhanced lipogenesis and suppressed lipid clearance.

Furthermore, the disruption of the gut-liver axis emerges as a pivotal amplifier of cadmium hepatotoxicity. Cadmium compromises intestinal barrier integrity, leading to endotoxin (e.g., LPS) translocation, while simultaneously inducing gut dysbiosis, reducing beneficial microbial metabolites such as SCFAs, and dysregulating bile acid metabolism (particularly via the suppression of FXR signaling). This inflammatory and metabolic signaling, derived from the gut, has been shown to significantly exacerbate hepatic inflammation, oxidative stress, and lipid accumulation.

Notwithstanding the significant advances that have been made, there are three key controversies that demand urgent resolution: changes in hepatic lipid homeostasis induced by chronic environmental cadmium exposure across different experimental models, the underlying basis of sexual dimorphism in susceptibility (likely estrogen-mediated), and the potentially synergistic hepatotoxicity arising from co-exposure with prevalent co-pollutants such as microplastics. The unresolved issues thus highlight the critical need for future research to be conducted using integrated multi-omics approaches (transcriptomics, epigenomics, metabolomics, metagenomics) combined with sex-stratified and chronic environmental dose exposure models so that the complex molecular networks can be fully delineated. Addressing these gaps is of the utmost importance for translating mechanistic insights into effective intervention strategies targeting cadmium-induced oxidative damage, inflammatory cascades, epigenetic modifications, and microbiota dysbiosis to mitigate the burgeoning global health burden of cadmium-induced MAFLD.

### Data and code availability

No data were used for the research described in the article.

## Acknowledgments

This work was supported by the 10.13039/501100007129Natural Science Foundation of Shandong Province (Grant No. ZR2022MH014) and WBE Liver Fibrosis Foundation (Grant No. CFHPC2025070).

## Author contributions

Ruilong Li: investigation and writing original draft. Xuemeng Wang: investigation and writing original draft. Wanwan Liu: writing original draft. Mingjie Song: writing original draft. Tao Zeng: review and editing and supervision. Cuili Zhang: conceptualization, review and editing, supervision, and funding acquisition.

## Declaration of interests

The authors declare that they have no known competing financial interests or personal relationships that could have appeared to influence the work reported in this article.

## Declaration of generative AI and AI-assisted technologies in the writing process

During the preparation of this work, the authors used DeepSeek AI in order to improve the readability and language of the article. After using this tool, the authors reviewed and edited the content as needed and take full responsibility for the content of the publication.
